# Experiences of young smokers in quitting smoking in twin cities of Pakistan: a phenomenological study

**DOI:** 10.1186/s12889-018-5388-7

**Published:** 2018-04-10

**Authors:** Kanwal Shaheen, Oyinlola Oyebode, Haleema Masud

**Affiliations:** 1 Al-Shifa School of Public Health, Al-Shifa Trust Eye Hospital, Rawalpindi, Pakistan; 2 Warwick Medical School, the University of Warwick, Coventry, CV4 7AL UK

**Keywords:** Cigarette smoking, Cessation, Experiences, Barriers, Failure, Young smokers, Unsuccessful attempts

## Abstract

**Background:**

Smoking is highly prevalent in Pakistan claiming the lives of over 100,000 individuals every year. A significant proportion of smokers (24.7%) make an attempt to quit each year but 97.4% fail to quit successfully. Little is known about the reasons for, and experiences of, failed quit attempts. This study was carried out to explore the experiences of young male smokers in quitting smoking in the twin cities of Pakistan

**Method:**

A qualitative study was carried out using a phenomenological approach in Rawalpindi and Islamabad. A total of 11 participants were interviewed. All study participants were male and had made at least one quit attempt. Study participants were a mix of smokers who failed to quit smoking, intermittent smokers and successful quitters. Streubert’s (1991) method of phenomenology was followed during data analysis.

**Results:**

The experiences of smokers while smoking “the smoking phase” have major effects on their journey towards quitting smoking. The smoking phase consists of three major stages: contact with initial smoking stimuli, the journey from first puff to enjoying smoking and then finally smoking becoming part of life. However, the journey towards quitting smoking is not as simple as the journey towards becoming a smoker. Instead, smokers get trapped in three overlapping cycles of smoking and quit attempts: smoking & forced quitting, smoking & intentional quitting, and smoking & intermittent smoking before successful quitting. Breaking the cycle is not easy in the presence of trapping factors (addiction, high availability, easy affordability, conducive social setup and low perceived risks of smoking). Three factors play a major role in breaking these cycles which are strong will power, continuous peer support and avoidance of smokers’ company.

**Conclusion:**

A young smoker, during his experience of quitting smoking gets entrapped in several overlapping cycles of smoking & quit attempts before successful quitting. There are known entrapping factors as well as factors which help in breaking these cycles. Targeted interventions are needed to facilitate smoking cessation among young smokers in Pakistan.

**Electronic supplementary material:**

The online version of this article (10.1186/s12889-018-5388-7) contains supplementary material, which is available to authorized users.

## Background

Pakistan is among the top 15 countries for burden of tobacco related morbidities and mortality [1]. There are almost 24 million tobacco users in the country, the majority being smokers [2]. Currently very few smokers (24.7%) make quit attempts in Pakistan as compared with other countries where 40%-50% of users try to quit every year [2–5]. The success rate of quitting is also low in the country, only 2.6% succeed [6]. International literature points out higher rates of successful quitting compared with Pakistan. In Brazil, only 42.1% of smokers experience a relapse after a quit attempt [3]. Findings from Italy showed a quit attempt rate of 40% among smokers with success rate of 8% at the first attempt [4]. In the United States, nearly 50% of smokers make at least one quit attempt in their life, the success rate is 3-5% for unaided attempts each year [5, 7]. A difference between the numbers of people willing to quit and those succeeding indicates a gap in the means available to achieve successful quitting [8].

Numerous epidemiological and qualitative studies have tried to understand this gap and identified factors responsible for successful or unsuccessful quitting. Successful quitting is dependent on higher socioeconomic status, older age, health status, quitting history, quit intentions, high taxation, awareness and use of assistance [9, 10]. Pakistani data shows that the majority of smokers are unable to attribute reasons for unsuccessful quitting, while others relate it to addiction, stress and peer pressure [6, 11, 12]. Abdullah and Husten have also highlighted several obstacles that could obstruct smoking cessation in low and middle-income countries like Pakistan including; poor healthcare systems, low awareness about health hazards of smoking among public, no smoking cessation policies and tobacco industry marketing strategies [13].

Use of qualitative studies is of paramount importance in understanding the complex process of quitting and for designing targeted interventions. Various qualitative studies and behaviour models (like the health belief model, theory of reasoned action, social cognitive theory and stages of change model) have been used to get a deeper understanding of smoking cessation processes and design smoking cessation services [14–19]. However, almost all of these studies are based on data from high income countries. Smokers in these countries live in a different cultural context and social environment where knowledge about smoking hazards is relatively high, effective national level policies exist to promote cessation, healthcare systems are supportive, and specialized cessation clinics and services are available. In contrast low and middle income countries like Pakistan have different cultural context, with health systems lacking specialized support for cessation and a dearth of context specific explanations on failed quit attempts [13]. A deeper understanding of the smoking cessation process is needed in these countries to guide targeted interventions. Phenomenological studies can best capture the lived experience of smokers and ex-smokers and elucidate the whole process of quitting from smokers’ perspectives [14]. This would not only highlight both barriers and facilitators of quitting, but also the need for specific support during different phases of quit journey. We carried out this study to capture the experiences of young smokers while quitting smoking in a low and middle income country, Pakistan, with the aim of elucidating the whole quitting journey. Such studies are relevant to policies in the Pakistani context where smoking cessation programs are in their infancy.

## Method

A phenomenological approach was used to explore the experiences of adult male smokers in quitting smoking in Pakistan. Phenomenology involves capturing the lived experiences of individuals who have experienced the phenomenon of interest, in this case quitting smoking. We used Streubert’s [20] procedural steps for carrying out phenomenology (attached as Additional file 1).

The study was conducted in Rawalpindi and Islamabad from April 2016 to July 2016. We recruited only males in the study considering the gender specific social context of smoking in Pakistan and other South Asian countries where smoking among men is common while less common and stigmatized among women [2, 21–23]. Other inclusion criteria for study participants were age up to 40 years and persons who were or had been smokers and had made at least one quit attempt. The age limit was applied considering the maximum health benefits gained by quitting before age 40 (evading more than 90% of the health risk), while quitting at age 50 would only result in evasion of 50% of the health risks [24, 25].

Study participants were recruited using a mix of purposive, snow ball and theoretical sampling techniques to fully elucidate the phenomenon. The starting point for selection of participants were the universities of Rawalpindi and Islamabad where young male smokers who had tried quitting at least once were invited to participate in the study using notice boards, social media and mobile phone based text messages. A total of five individuals showed interest within ten days of the invitation. However, two refused to participate in the study after knowing details about interview method and recording. One of the three selected participants suggested two more smokers meeting the inclusion criteria. The next four participants were selected using theoretical sampling technique based on emerging findings to complete the description of phenomenon i.e., smoking cessation. This made a mix of five current smokers, four intermittent smokers and two successful quitters. Intermittent smokers were defined as those who were not daily or regular smokers [26–28] while successful quitters were those who had been abstinent from cigarettes for one year or longer [29, 30]. This mix was required to fully elucidate the lived experiences of quitting smoking among young adults and identify what ultimately leads to successful quitting.

We interviewed a total of 11 men for this study, phenomenology usually requires a sample of 1-10 for full description of phenomenon [31]. Interview date, time and venue were based on mutual agreement of the participants and researcher. An in-depth interview guide was used to collect information from study participants as per Kvale’s recommendations [32]. Interviews were taken in participants’ native language, Urdu, to enable them to feel comfortable to talk and express themselves fully. Each interview was audio recorded by mobile (mp3). Interview duration ranged from 30 to 71 min.

We also used other data collection methods like a log book, field notes and a peer debriefing journal. An interview log was maintained to record socio-demographic details of participants and notes during interviews. Field notes were also taken to record the expression, emotions and body language of the participant. These were used at analysis step to guide the interpretations of audio recordings. The peer debriefing journal was maintained to record debriefing sessions with peers after each interview and also during analysis stage. The notes in the journal helped in expansion of interview guide for later interviews, guided the whole analysis process and model development. One focus group discussion was conducted after the main data analysis, to validate the model and record second hand information regarding experiences of young smokers in quitting smoking. This is called shadowed data technique [33]. Seven participants including health professionals, PhD scholars and students were recruited for focus group discussion. These participants were not smokers but had smokers in their families, social circle and were health professionals.

Data analysis was done side by side with data collection. All interviews were transcribed using online time stretcher software. The transcribed interviews were read many times before starting the analysis. Seven steps as per Streubert [20] method of phenomenology were followed during data analysis. Intuiting was the first step in data analysis which involved development of initial perceptions about the phenomenon (experience of quitting) by immersing in the descriptions of the experience [20]. Intuiting was begun after first interview. Participants’ description of their experience was listened to attentively, their body language was observed intently and vocal intonations were carefully noted. Audio recorded interviews were listened to and transcripts were read several times to ensure quality of intuiting. After intuiting, the next step was phenomenological analysis, formulation of codes and common themes to reach more abstract essences [20]. OpenCode 4.02 software was used for coding. All codes, themes and essences were assigned manually. An example of a journey from transcript to essences is given in the Additional file 2 attached. Peer debriefing and imaginative variation was used in the later stages of analysis to identify relationships between and patterns across essences. During the iterative process of intuiting, analyzing, and apprehending essential relationships, participants were contacted for their feedback on formalized description of phenomenon to develop an accurate and thorough understanding of the phenomenon. These follow up interviews provided opportunities to identify whether codes, essences, or relationships across essences required modification. A summary of the formalized description of phenomenon was shared with interested participants for feedback. Five of these participants provided feedback and agreed that the findings were close to the discussions during interviews.

Ethical approval for research was taken from Institutional Review Board (IRB) of Al Shifa Trust Eye Hospital and written informed consent was obtained from all participants. Confidentiality and anonymity was assured to each participant.

## Results

A total of eleven individuals participated in the study. All participants were male with mean age 26.9 (SD = 4.51) years ranging from 22 to 40 years. All participants were unmarried except one current smoker and one successful quitter. Table 1 lists the socio-demographic information of participants. Five participants were current smokers, four were intermittent smokers and two were successful quitters. One of the successful quitters had quit smoking 15 years previously and the second one had quit 2 years previously. Intermittent smokers reported smoking one or two cigarettes per week on average and occasionally exceeding this limit while current smokers reported 1-20 cigarettes per day. Smoking duration ranged between 2 and 11 years; the average time since smokers started smoking was 6.72 years. Most participants had initiated cigarette smoking during adolescence (mean age was 17 years) or during their college time.

**Table 1:** Demographic and Smoking Characteristics of Participants

Sr. No.	Age (years)	Occupation	Socio-economic status	Smoking status	Age (smoking initiation)	Smoking duration	Quitting duration	No. of cigarettes used
1	22-25	Student	Lower middle class	Current Smoker	16 years	6 years	N/A	1-20/day
2	26-30	Student	Lower middle class	Current smoker	17 years	9 years	N/A	1-10/day
3	26-30	Unemployed	Lower middle class	Intermittent smoker	21 years	7 years	N/A	On average 1-2 per week, but occasionally smoke more than that
4	22-25	Student	Upper middle class	Current smoker	17 years	8 years	N/A	1-20/day
5	22-25	Employed	Upper middle class	Current smoker	20 years	2 years	N/A	1-10/day
6	31-35	Employed	Upper middle class	Current smoker	22 years	13 years	N/A	15/day
7	22-25	Student	Upper middle class	Intermittent smoker	18 years	4 years	N/A	On average 1-2 per week, but occasionally smoke more than that
8	26-30	Employed	Lower middle class	Successful quitter	17 years	12 years	2 years	N/A
9	31-35	Employed	Lower middle class	Successful quitter	13 years	7 years	15 years	N/A
10	22-25	Student	Upper middle class	Intermittent smoker	17 years	8 years	N/A	On average 1-2 per week, but occasionally smoke more than that
11	26-30	Student	Lower middle class	Intermittent smoker	17 years	9 years	N/A	On average 1-2 per week, but occasionally smoke more than that

All the study participants had made at least one quit attempt while some had made several attempts. Most of the participants used cardamom and tea as alternatives to cigarettes during quit attempts. Two participants switched to snuff to lessen the craving for cigarettes. One of the participants used nicotine gum as a smoking cessation aid, he had heard about it from a commercial but he reported that it was not helpful. He had also consulted a doctor for cessation support who had suggested that he try a healthy diet as an aid to cessation but which he also found to be unhelpful. None of the participants had any idea about smoking cessation centers in Pakistan. None of the participants reported any diagnosed disease associated with smoking.

### Experience of quitting smoking

**Fig. 1:**
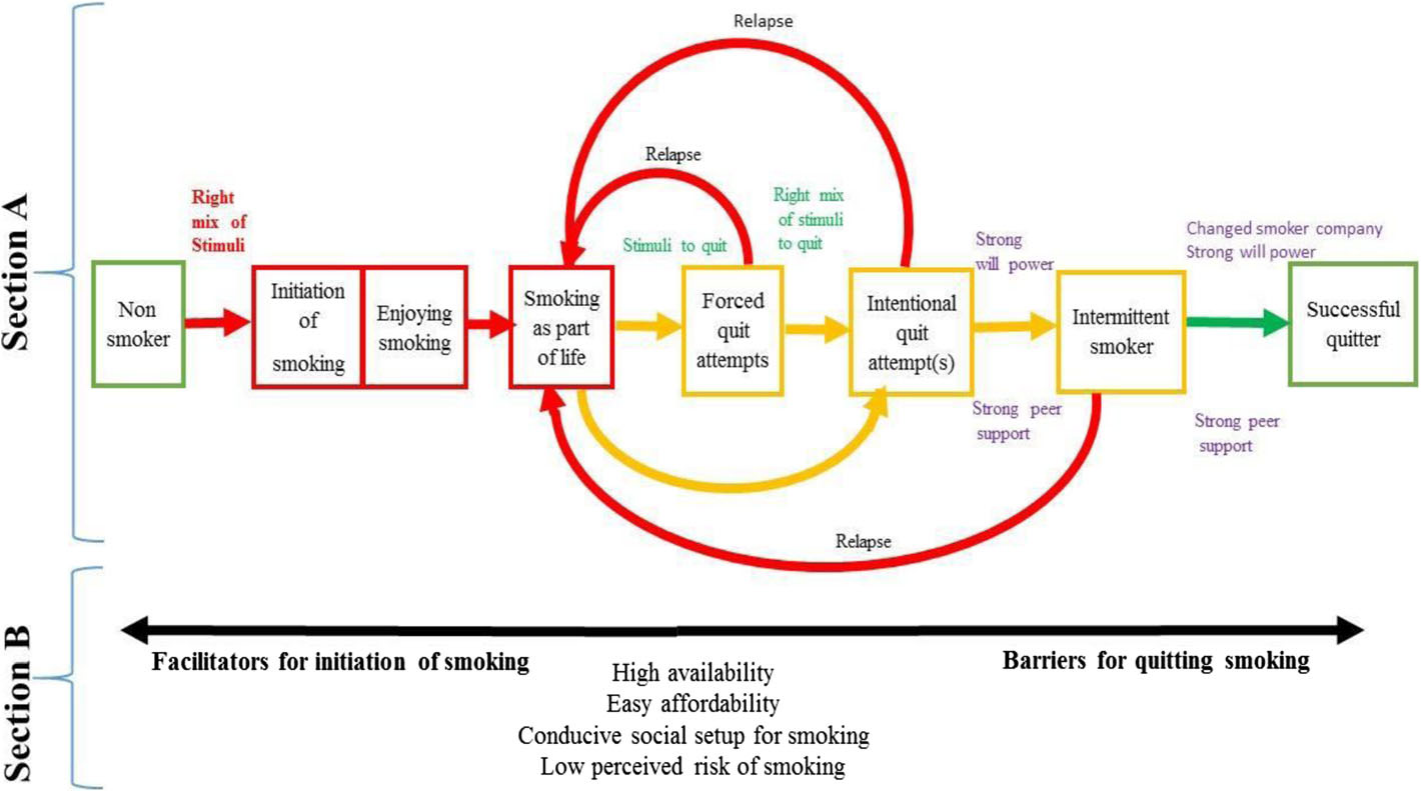
Experiences of quitting smoking among ‘young’ male smokers

After rigorous analysis of all data, lived experience of smokers were condensed into a model comprising of two sections (Fig. 1). The first section consists of different stages through which a smoker passes during the journey toward quitting. The second section encompasses a list of factors that are consistent throughout the smoking journey. These factors act as facilitators during smoking initiation and become barriers to quitting smoking.

#### Section a: Smoking phase and quitting phase

A smoker passes through several stages during his journey to becoming an ex-smoker. For young smokers, experiences of quitting smoking are deeply embedded in the experiences of starting smoking and living a smoker’s life. They compare their feelings and experiences of quitting with the feelings and experiences at the time when they had started smoking and were living a smoker’s life.

The whole experience of the smoking journey can be summarized in two phases; the smoking phase and the quitting phase, each comprising of different stages. The movement from one stage to the next stage depends on presence of pull factors and absence of entrapping factors.

### Smoking phase

The smoking phase starts when a non-smoker gets exposed to multiple stimuli that motivate them to smoke. They cannot resist the urge and start smoking. After the first puff, young smokers learn proper smoking techniques and start to enjoy it. It then becomes a major part of their life. The factors listed in part B of the model (Fig. 1) act as facilitators in uptake of a smoking habit. The smoking phase can be described as consisting of three major experiences or stages: first exposure to stimuli that promote smoking, the journey from first puff to enjoyment of smoking and then finally smoking as part of their lives.

### Exposure to stimuli that promote smoking

Becoming a smoker was expressed by participants as entering the world of fantasy. The non-smoker entered this world for pleasure and well-being and this entry was based on exposure to a mix of stimuli including curiosity, peer pressure, trend and feeling a need to start smoking (Fig. 1).

Curiosity was found to be a major reason that motivated individuals to start smoking. Living, working or socializing with smokers had made the non-smoker curious about smoking. As participants expressed;

“*Whenever I used to see a person smoking, I used to get curious… I wanted to know what is smoking and how it feels to smoke.…, the way they inhale smoke and then the way they blow it out*...” (Participant A)

Participants also pointed out peer pressure as a stimulus which promoted smoking. Non-smokers felt they needed to start smoking to be friends with smokers and to socialize with them.

“*My friends were smokers, they were my community and I used to feel left out as a non-smoker when they were smoking. Then, I started smoking with my friends*”…. (Participant C)

Participants declared cigarette smoking as fashionable, stylish and trendy which also acted as a stimulus for them to start smoking.

“*Cigarette ads on TV were fascination for me, smoking was depicted as an amazing thing in commercials, as smokers were shown climbing on mountains…. it was something very cool to me*”… (Participant E)

Curiosity, peer pressure and smoking as a trend had instilled the need to smoke cigarettes as revealed by participants. Participants divulged that they had started smoking by their own will.

“…*it was like… as if I felt an urge from inside to smoke cigarettes…you know the feeling when someone is hungry and has food in front of him, he feels a strong urge to eat that food… so the way smoker was smoking and enjoying captivated me to try one*” … (Participant Q1)

### Journey from first puff to enjoying smoking

**Fig. 2:**
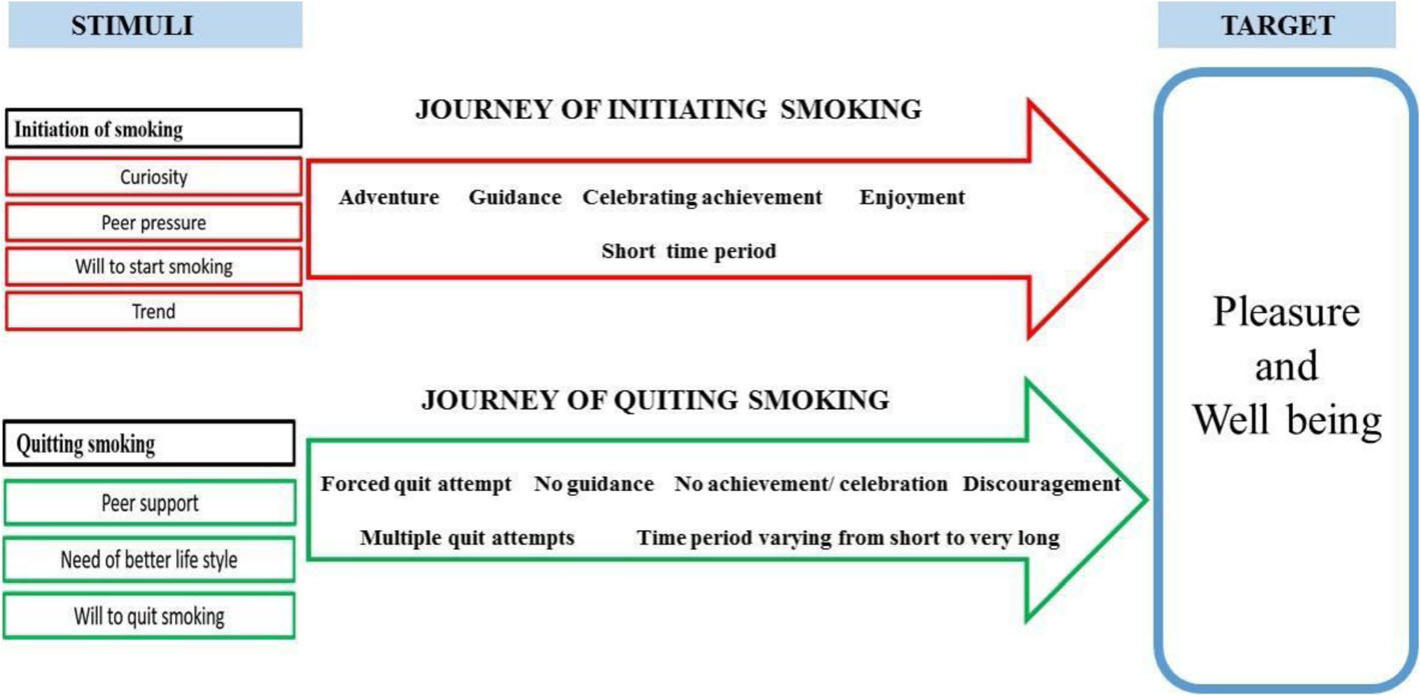
Comparative journey of initiation and quitting smoking

Participants revealed that when they entered the world of smoking, at first, they did not know how to smoke. They started puffing and then were facilitated by their smoker friends about the actual technique of smoking. Their friends had fully guided them towards the right smoking technique. After the first successful smoking attempt, they celebrated their achievement and they remember it as a great memory which had given them pleasure and improved their well-being (Fig. 2).

Participants were taught that inhaling the smoke of a cigarette is real smoking.

“*My friend told me how to actually inhale cigarettes smoke…. He told me that what I was doing was not real smoking, it's puffing…he explained to me.… soon after taking the cigarette in the mouth make a sound "sseee" with your tongue only in this way you can actually inhale smoke'… get the real effect of cigarette*”….. (Participant C)

After learning the actual technique of smoking, participants really enjoyed it and celebrated their achievement.

“*When I smoked in the right way… I looked at my friend proudly… in a way…to show that… now I know how to smoke and how it feels. It was amazing*” (Participant A)

Among good memories, the most unforgettable memory for some participants was the feeling they got when smoking that first cigarette. Participants expressed that in pursuing that first feeling they actually became a regular smoker.

“*First feel…. My senses were gone, I felt as if I had no control over me. It really fascinated me. It had brought me out of tension. So, in pursuing that first feel I started smoking daily*”…. (Participant C)

Participants reported that smoking cigarettes had always given them enjoyment and relieved everyday stress from their lives.

“*Cigarette is a felicity, bliss and happiness. I mean it is an enjoyment for me*”….. (Participant F)

### Smoking as a part of life

The participants reported that smoking was started as fun but gradually it became a necessity, a daily habit and an important part of their life.

“*I had started it (smoking cigarettes) as fun, then it became my habit…now it is difficult for me to quit*”… (Participant J)

The participants also pointed out that their smoking habits are paired with their everyday activities.

“*After eating the meal, I feel a strong urge to smoke, just do nothing but to smoke and if I do not smoke, I get a headache, a feeling as if something is missing and I have lost something. I feel like my meal is incomplete without cigarette*” …. (Participant A)

Participants expressed that they were so addicted to smoking that it became an important routine task of their life.

“*For me, cigarette smoking is a routine and a habit*”…… (Participant D)

Smokers did not view cigarettes as tobacco ie: a substance, they expressed that cigarettes could be companions.

“*Cigarette for me is a way to spend time, whenever I have nothing to do, I smoke cigarettes…. cigarette is my companion of loneliness*”… (Participant C)

The participants pointed out that they use smoking as a coping strategy for the emotional problems of everyday life.

“*Whenever I feel like crying or I am stressed, cigarettes relax me to a great extent, they divert my attention*”…. (Participant F)

Participants developed a defensive attitude towards smoking at this stage and they were in denial about the serious harmful aspects of smoking.

“*What’s bad in smoking? We are not doing anything bad. No, smoking is not a bad thing*” (Participant D)

### Quitting phase

The quitting phase starts with a quit attempt as a result of some stimuli hitting smokers just like the stimuli hit them for smoking initiation. Smokers move through different stages namely forced quit attempt(s), intentional quit attempt(s), becoming an intermittent smoker and then a successful quitter. The journey from getting in contact with stimuli to successful quitting was not reported to be as smooth and simple as was the case in journey of smoking initiation. Instead, smokers get trapped in three overlapping cycles of: smoking & forced quitting, smoking & intentional quitting and smoking & intermittent smoking before successful quitting. Breaking the cycle is not easy in the presence of trapping factors (addiction, high availability of cigarettes, easy affordability of cigarettes, a conducive social setup for smoking and low perceived risks of smoking). Three factors were reported to play a major role in breaking these cycles: continuous peer support, strong will power and avoidance of smokers’ company by young smokers and quitters.

Participants shared that quitting smoking was not a simple task. It was expressed as more than leaving a habit, in fact sacrificing a part of one’s life. It was leaving a companion of loneliness, sacrificing a buddy through meddlesome events, parting from one’s stress reliever, and changing one’s everyday routine.

### Cycle of forced quit attempts and smoking

Participants reported that their first quit attempt was not their personal choice rather a forced decision. Smokers expressed that their families compelled them to quit smoking emotionally or forcefully.

“*When my family came to know about my smoking, they used the typical way of beating… they had beaten me, forced me to quit and they had taken the promise from me not to smoke again”…* (Participant A)

Forced quit attempts were never successful in the experience of our participants, no matter for how long the forced quit attempt lasted they always had a relapse and returned to their normal smoking habit. The major reason was the lack of personal will.

*“I am not ready to quit smoking. There is no other reason just that I do not want to quit”*…. (Participant D)

Another important feature of this stage was that the family pressure was not continuous. It started with high intensity but then family members got used to the person as a smoker and believed that quitting was impossible.

*“As I grew older, my family accepted my smoking… my mother sometimes asks me to quit but my other siblings declared me independent in this regard. They are like… do whatever you want to do”…* (Participant A)

### Cycle of intentional quit attempt and smoking

The cycle of smoking and forced quit attempts breaks when a right mix of stimuli hit smokers and instils a desire in them to quit. These stimuli were comparable to the ones reported in smoking initiation, however less intense and fewer in number (Fig. 1). The ultimate target was to improve well-being by quitting smoking. These stimuli were peer support, a desire for a better lifestyle and a wish to quit smoking (Fig. 2). When these stimuli were encountered by a smoker, he started his intentional journey towards becoming a non-smoker. Our data suggests that continuous presence of these stimuli at strong intensity in the smokers’ lives was crucial for successful quitting. Most of the participants faced relapse and were trapped in this cycle. The previous experiences of smokers at this stage were highly crucial in influencing whether he would get trapped in this cycle of succeed in moving on.

Participants at this stage expressed that they had weak will power, weak peer support and strong barriers which were the addiction, high availability, easy affordability, conducive social setup for smoking and low perceived risk of smoking. Hence, relapse occurred at this stage for most of the participants (Fig. 1). Participants shared varied durations of temporary success before they resumed smoking i.e. from less than one day to seven months. They reported that they had experienced several such intervals.

Participants made intentional quit attempts with weak will power and weak support from peers. They did not get guidance, as they got when initiating smoking; instead, they were discouraged by peers (Fig. 2).

*“When I tried to quit smoking, no one cooperated. If I tried to ask someone to help me, instead of helping, they taunted that why I had started smoking …. Even my friend, just for formality, said, ‘yes, it is good to quit smoking but dear friend you cannot’ (quit smoking)*” (Participant C)

In our findings, some smokers had made even more than 25 attempts to quit but they were not successful. This was the toughest stage of the quitting phase, as in spite of his own wish to quit, he failed to do so. Our findings revealed that here in this stage, a smoker’s willpower alone, was not strong enough to quit smoking.

*“Everyone told me that I can quit by will power. I think, may be my will power is weak and only those who have strong will power can do it”….* (Participant C)

### Cycle of intermittent and regular smoking

Participants could break the cycle of intentional quit attempts & smoking when they got strong peer support and thereby developed strong will power. After developing strong will power and receiving strong peer support, participants had made quit attempts, they were successful in the sense that they had reduced number of cigarettes and were smoking occasionally. However, our participants could not all completely quit, some reduced their habit to smoke one cigarette in a week or smoked off and on and became intermittent smokers.

*“I reduced the number of cigarettes by will power, for example from 12 cigarettes to 2 cigarettes but I could not completely quit smoking”…* (Participant C)

Intermittent smokers could relapse to smoking in the following situations:

***Stressful life events***; when these intermittent smokers were sad, depressed, stressed, worried or had no other solution or support, they restarted smoking.

*“Often when I am in depression, as now-a-days… normally people get depressed two or three times a day. So, I restarted smoking whenever I had this depression phase”…* (Participant C)

***Smokers’ Company***; participants expressed that when they were in the company of smokers they smoked or sometimes relapse occurred upon staying with them for long time.

*“My close friends are smokers…. It is very difficult for me to quit smoking”…* (Participant C)

***Free time and loneliness***; for a quitter, the most tough or challenging thing was free time. It was a big hindrance in maintaining quitting, when a smoker had nothing to do, he again started smoking as cigarettes made him feel busy and gave him the feeling of being in company.

*“I started smoking again because most of the time I had to be alone. I did not have anything to do. I did not have much workload. So, I restarted smoking”…* (Participant D)

Movement from this stage to next stage was dependent on strong will power and peer support and absence of free time, loneliness, stressful life events and smokers’ company.

### Successful quitting

Successful quitters among our participants revealed that strong will power, continuous peer support and no longer seeking smokers' company helped them in maintaining quitting and decreased the chance of relapse. However, the entrapping factors (Section B, Fig. 1) made this transit very difficult and very few smokers reached the stage of successful quitting.

*“In the start, I had quit smoking for hours, and then I had quit it for days. Then I changed my smoker friends, and finally, I have quit smoking forever. I think if you get a good companion, quitting smoking can be easy…. I got that companion, who never let me feel alone, talking to that friend never makes me feel time is not passing…. When I am with my friend, I never feel craving for cigarette”*… (Participant Q1)

#### Section B: Facilitators in initiation and barriers during quitting smoking

Participants shared that the experiences of the quitting journey were not smooth and resulted in entrapment in different cycles because of some external factors. These factors had been acting as facilitators at the start of their smoking journey. However, these facilitators were transformed into strong barriers later quitting smoking. They acted as an entrapping force for smokers to keep them in the cycles. These entrapping factors were namely: high availability, easy affordability, conducive social setup for smoking and low perceived risks of smoking.

### High availability

Cigarettes are easily available everywhere which makes non-smokers easily get attracted towards cigarettes. Seeing everyone smoking around, cigarette availability in almost every shop makes it very easy for non-smoker to start smoking. Participants had highlighted this issue;

“*Cigarette availability everywhere is the big reason of smoking. It's available in every shop… almost everywhere, this is the reason everyone is smoking… from teenagers to older ones”…* (Participant Q2)

High availability acted as a strong barrier in quitting smoking.

“*If you want to quit (cigarette smoking), it is difficult… as it (cigarette) is available everywhere”…* (Participant A)

### Easy affordability

Another strong stimulator in smoking initiation and strong barrier in quitting smoking mentioned by participants was easy affordability. Participants mentioned that in Pakistan cigarettes are easily affordable at low cost.

*“This is the biggest issue… I think cigarettes in Pakistan are very cheap and available everywhere. When I was in Saudi Arabia, cigarettes’ availability was very low and cigarettes were much expensive. Their least costly cigarette, in Pakistan is the most expensive one”….* (Participant E)

### Conducive social setup for smoking

Social acceptability was a big challenge as well. Cigarette smoking was not stigmatized in society as expressed by participants. It is quite acceptable behavior especially when you cross adolescence and when it is common in a family.

*“On my elder brother’s wedding, all cousins were gathered. We smoked cigarettes together and we did it throughout the night. It was fun and even our families did not mind it”…* (Participant B)

People compared cigarettes with other addictive substances which were labeled as bad and stigmatized but cigarettes as normal. So, this conducive setup promoted smoking initiation and acted as a strong barrier for a quitter in quitting smoking. As a participant said:

*“No, smoking is not bad; it does not harm you as such as compared to other things. If you say only smoking is bad, you are wrong as there are many other things which are bad and we do not pay attention to them”…* (Participant D)

### Low perceived risks of smoking

Participants perceived smoking as having low risks. This perception facilitated them to start smoking and acted as a strong barrier while planning to quit smoking.

*“Well, I have seen a smoker, who is smoking for 8 years; he has not got cancer yet. I have many other examples as well… no one has seen a smoker yet who get cancer within 2 years or within 4 year…. so as a smoker I believe, it does not harm, it will not cause you cancer or any other major ailment”…* (Participant C)

## Discussion

The present research aimed to explore the experiences of young smokers in quitting smoking using the phenomenological approach. The main findings of the study suggest that quitting smoking is a complex journey, where quitting experiences are deeply embedded in the experience of starting smoking. A smoker gets entrapped into three overlapping cycles of smoking and quit attempts: smoking & forced quitting, smoking & intentional quitting, and smoking & intermittent smoking before successful quitting at a young age. Breaking the cycle is not easy in the presence of trapping factors (addiction, high affordability, easy availability, conducive social setup and low perceived risks of smoking). Three factors play a major role in breaking these cycles which are strong will power, continuous peer support and avoidance of smokers’ company. Our model discusses smoking cessation as a stage wise process like the trans-theoretical model which explains a behavioral change in terms of different stages (Pre-contemplation, contemplation, determination, action, maintenance/relapse/recycle and termination) [17]. It also highlights factors external to individual control like affordability and availability.

Our findings suggest that smokers compare their quit journeys to their experiences when they first entered the smoking world. Entry into smoking is stimulated by curiosity, peer pressure and fashion. In contrast, similar factors to stimulate quitting are weak or non-existatnt. Previous studies have shown that smokers attempt to quit due to social pressure, intrinsic health concerns and stigmatization [34–37] whereas our study showed low perceived health risks, lack of stigmatization, and a socially conducive set-up for smoking in Pakistan. This may explain why fewer users attempt to quit (24.7%) in the country compared to other countries where almost 40%-50% users try to quit [2–5]. As smoking is reported to spread amongst groups of friends in Pakistan, using social groups in cessation may be of great help. Evidence shows that such groups provide an environment where people may find the support necessary to overcome the challenge of quitting tobacco addiction, share their experiences and difficulties [38, 39]. Smoking cessation programs should also consider the key points of fashion and peer pressure while designing interventions for young people. Bringing smoking cessation into the media limelight and making it fashionable could help motivate smokers to quit. Likewise using the social capital of family and peers to promote smoking cessation. Participants highlighted that their initial attempts to quit were always based on family or friends’ pressure but the pressure was not continuous and they had relapsed. Family pressure is an established triggering factor for quit attempts [34, 40] and has been used for smoking cessation in different high income settings [41–43]. However, results of such interventions are not very promising [43]. Using family based interventions is an unexplored potential area in Pakistan where family bindings and structures are strong.

Our study emphasizes that smokers’ entry to the smoking world is fully guided and facilitated by friends. Wang, Gjengedal and Larsen also presented similar findings that smoker friends properly train new smokers on how to smoke [34]. This is in sharp contrast with the quit journey captured in this study, where nobody guides smokers wishing to quit and there is possibility of discouragement as well. Smokers in Pakistan highlighted that there was no training, guidance or proper treatment to support cessation. Similar findings of lack of support, being alone in quit attempts, lack of role models and taunting comments from peers have been discussed in other studies as reasons for unsuccessful quit attempts [44]. Many countries have introduced behavioral training and pharmacologically or professionally mediated interventions to assist smoking cessation for different target groups [45–47]. There is a need to introduce easily accessible and affordable smoking cessation services in Pakistan especially for young smokers who are not suffering from chronic diseases. An immediate support for smoking cessation becomes more important for young smokers, as unassisted efforts lead to continuous relapse (entrapment in cycles or quitting and relapsing) and discourage further attempts as found in our study and other literature [48, 49].

We found that smokers can break the cycle of intentional quit attempts & smoking when they develop strong will power. Other studies have also reported will power as a key variable in quitting, a strategy to counteract cravings and a personal quality or trait fundamental to quitting success [50–52]. Theory of reasoned action/planned behavior states this as perceived control which is based on positive attitude (individual’s belief) and subjective norms [16] while social cognitive-behavioral theory name it ‘self-efficacy’ [18]. Will power as expressed by our participants is not something static; instead, it builds with time by intrinsic and extrinsic motivation like peer support. Continuous peer support provides an alternative to cigarettes by providing psycho-social support and substituting all such values attached to smoking. Our findings are supported by other studies which showed using this support concept as an intervention to help smokers to quit [41, 49, 50, 53].

Breaking the cycle of quitting and relapsing is not easy in the presence of trapping factors such as addiction, high availability, easy affordability, conducive social setup for smoking and low perceived risks of smoking. These results showed convergence with studies which highlighted nicotine addiction, ease of purchase and availability of cigarettes as a hindrance in quitting smoking [14, 54, 55]. Easy access to cigarettes or availability everywhere was also discussed in studies as making quitting difficult and causes relapse more frequently [7, 54]. Cigarettes are not only available everywhere but also at very low cost in Pakistan. The low price of cigarettes makes them readily affordable for everyone. Smokers can also purchase one or two cigarettes from open packs which decrease theirs cost further. There are varieties of cigarettes available at a range of prices in Pakistan. The minimum price of a cigarette is US$0.15 and the maximum is $1.3 with tax $0.09 [56]. If we compare this price with other countries, in Saudi Arabia a cigarette costs $1.68 to $22.4, in the US a cigarette costs more than $14 with tax of $4.3 per 20 cigarette pack and in the UK a pack/a cigarette costs $12.94 with tax $9.51 [57, 58]. These figures show Pakistan offers cigarettes at the lowest price. This hinders quitting smoking. Similar findings are shown in a study which states ease of purchase of cigarettes makes quitting difficult and creates trigger for relapse [7, 55]. Moreover, the conducive social setup which is a big barrier in a country like Pakistan in contrast to high-income countries where smoking is becoming more stigmatized [35, 37]. Pakistan needs to strengthen its overall tobacco control program.

Our study represents only the experience of urban-dwelling young male smokers as we conducted interviews with those who are living in urban areas. Moreover, we could not cover opinions of very poor and elite class smokers; who may have different experiences. Results of the study need to be cautiously generalized, as it is based on a small sample of young smokers below 40 years of age. We found it challenging to categorize smokers as regular, intermittent or quitters per se as participants had shared multiple movements in and out of these stages. Some of the participants had labelled themselves, as well as others, as successful quitters although they had actually either decreased their smoking frequency or were smoking on an intermittent basis.

## Conclusion

The journey of quitting smoking is quite complex and is deeply embedded in the experience of starting smoking. The stimuli which cause initiation of smoking are important in quitting too. A young smoker, during his experience of quitting smoking gets entrapped into several overlapping cycles of smoking & quit attempts before successful quitting. Being entrapped in such attempts in itself discourages successful quitting. Breaking the cycle is not easy in the presence of trapping factors (addiction, high availability, easy affordability, conducive social setup for smoking and low perceived risks of smoking). Three factors play a major role in breaking these cycles which are continuous peer support, strong will power and avoidance of smokers’ company.

Considering the complexity of smoking cessation phenomenon, multi-faceted smoking cessation programs are required in Pakistan. Our findings highlight the need to target fashion and peer pressure as stimulants to promote smoking cessation. Media campaigns could be launched to make non-smoking status fashionable. Smoking should also be banned in TV dramas and movies; where it is often shown as a stylish way to cope with stress. An overall change in social norms is desired making smoking less of a normal behavior. As smoking behavior is adopted in groups, group interventions for young smokers may be successful. This study has highlighted peer support as key factor to quit smoking. Peer support systems have been successfully used for quitting smoking in other countries like the US and UK [45–47]. Peer support systems for young smokers should be developed in colleges, universities and communities to direct young ones towards constructive and productive social habits and positive common goals. In addition a “no smoking day or week” could be celebrated where ex-smoker can share their success story of quitting. There could also be motivational speeches of successful quitter with young smokers organized by universities and colleges. Pakistan can learn from the UK where smoking cessation rates have increased from below 14% to almost 20% due to developing a conducive environment for quitting and providing wide range of quitting methods like social support, motivational campaigns, and ban on attractive imagery on cigarette packs etc. [45].

Our research also highlights that smoking cessation is a physically, psychologically and emotionally charged process which requires expert advice and therapy as well as continuous peer support and will power. Our findings suggest lack of cessation services for young smokers in Pakistan. There is urgent need to make such context specific services available. Moreover the role of families and friends need to be explored in designing smoking cessation services for young smokers in Pakistan.

## Additional files

Additional file 1: Streubert’s procedural steps of phenomenology. Methodological steps followed during this study adapted from ‘Streubert’s procedural steps of phenomenology’. (DOCX 13 kb)

Additional file 2: ‘Example of a part of the analysis process. An example of the analysis process: From transcript to essence. (DOCX 13 kb)

## References

[1] World Health Organization (WHO): Fact Sheet on Tobacco control in Pakistan [2015]. Retrieved from http://www.who.int/tobacco/about/partners/bloomberg/pak/en/. Accessed 15 Jan 2018.

[2] Global Adult Tobacco Survey (GATS). Fact Sheet Pakistan 2014. 2014. Retrieved from http://www.who.int/tobacco/surveillance/survey/gats/pakfactsheet.pdf.

[3] Ferreira SAL, Teixeira CC, Corrêa APA, et al. Reasons that make individuals in a higher education institution to become or not to become smokers. Rev Gaúcha Enferm. 2011;32(2):287-93. 10.1590/s1983-14472011000200011 21987989

[4] D’Argenzio A, D’Argenio P, Ferrante G, et al. 40% of smokers try to stop smoking, only 8% succeed in. Epidemiol Prev. 2011;35(5-6):362. 22166786

[5] Centers for Disease Control and Prevention (CDC). Quitting smoking among adults-United States, 2001-2010. Morb Mortal Wkly Rep. 2011;60(44):1513-9. 22071589

[6] Irfan M, Haque AS, Awan S, et al. Reasons of failure to quit smoking: a cross sectional survey in major cities of Pakistan. Eur Respir J. 2014;44(Suppl 58):P4466.

[7] Jarvis MJ, McIntyre D, Bates C, Foulds J. Effectiveness of smoking cessation initiatives. Efforts must take into account smokers' disillusionment with smoking and their delusions about stopping. BMJ. 2002;324:608. PMC1122522 11884332

[8] Rafful C, García-Rodríguez O, Wang S, Secades-Villa R, et al. Predictors of quit attempts and successful quit attempts in a nationally representative sample of smokers. Addict Behav. 2013, 2013;38 10.1016/j.addbeh.2012.12.019. 10.1016/j.addbeh.2012.12.019 PMC3578080 23380497

[9] Scollo MM, Winstanley MH (2012). Tobacco in Australia: facts and issues.

[10] Lee C, Kahende J. Factors associated with successful smoking cessation in the United States, 2000. Am J Public Health. 2007;97 10.2105/AJPH.2005.083527. 10.2105/AJPH.2005.083527 PMC1931453 17600268

[11] Malik AK, Chaudhry A, Karamart A. Cigarette smoking and health care professionals at Mayo Hospital, Lahore, Pakistan. J Pak Med Assoc. 2010;60(6):509-12. 20527661

[12] Qidwai W. Barriers to smoking cessation: results of a survey among family practice patients. Middle East J Fam Med. 2004;5(5). Retrieved from https://ecommons.aku.edu/pakistan_fhs_mc_fam_med/146.

[13] Abdullah ASM, Husten CG. Promotion of smoking cessation in developing countries: a framework for urgent public health interventions. Thorax. 2004;59 10.1136/thx.2003.018820. 10.1136/thx.2003.018820 PMC1747072 15223875

[14] Jesus MCP, Silva MH, Cordeiro SM, Kortchmar E, Zampier VSB, MAB M. Understanding unsuccessful attempts to quit smoking: a social phenomenology approach. Rev Esc Enferm USP. 2016;50 10.1590/S0080-623420160000100010. 10.1590/S0080-623420160000100010 27007423

[15] Girma E, Assefa T, Deribew A. Cigarette smokers' intention to quit smoking in Dire Dawa town Ethiopia: an assessment using the Transtheoretical model. BMC Public Health. 2010;10:320. 10.1186/1471-2458-10-320. 10.1186/1471-2458-10-320 PMC2889871 20529337

[16] Ajzen I. The theory of planned behavior. Organ Behav Hum Decis Process. 1991;50:179–211. 10.1016/0749-5978(91)90020-T.

[17] Prochaska JO, DiClemente CC (1984). The Transtheoretical approach: crossing traditional boundaries of therapy.

[18] Bandura A. Social-cognitive theory: an agentic perspective. Asian J Soc Psychol. 1999;2:21-41. 10.1146/annurev.psych.52.1.1 11148297

[19] Becker MH (1974). The health belief model and personal health behavior.

[20] Streubert HJ. Phenomenological research as a theoretic initiative in community health nursing. Public Health Nurs. 1991;8(2):119-23. 10.1111/j.1525-1446.1991.tb00655.x 1924105

[21] Nisar N, Qadri MF, Fatima K (2007). A community based study about knowledge and practices regarding tobacco consumption and passive smoking in Gadap town. Karachi J Pak Med Assoc.

[22] Gupta PC, Ray CS (2003). Smokeless tobacco and health in India and South Asia. Respirology.

[23] Masud H, Oyebode O. Inequalities in smoking prevalence: a missed opportunity for tobacco control in Pakistan. J Public Health. 10.1093/pubmed/fdx044. 10.1093/pubmed/fdx044 28505324

[24] Jha P, Ramasundarahettige C, Landsman V (2013). 21st-century hazards of smoking and benefits of cessation in the United States. N Engl J Med.

[25] Sakata R, McGale P, Grant EJ (2012). Impact of smoking on mortality and life expectancy in Japanese smokers: a prospective cohort study. BMJ.

[26] Schane R, Glantz S, Ling P. Nondaily smoking: an increasingly prevalent pattern. Arch Intern Med [2009]. [169] 10.1001/archinternmed.2009.315. 10.1001/archinternmed.2009.315 PMC4350771 19858429

[27] Husten CG. How should we define light or intermittent smoking? Does it matter? Nicotine Tob Res. 2009;11 10.1093/ntr/ntp010. 10.1093/ntr/ntp010 PMC2658911 19246425

[28] DiFranza JR, Savageau JA, Fletcher K, et al. Symptoms of tobacco dependence after brief intermittent use: the development and assessment of nicotine dependence in Youth-2 study. Arch Pediatr Adolesc Med. 2007;161(7):704-10. 10.1001/archpedi.161.7.704 17606835

[29] Hughes JR, Keely J, Naud S. Shape of the relapse curve and long-term abstinence among untreated smokers. Addiction. 2004;99:29-38. 10.1111/j.1360-0443.2004.00540.x. 10.1111/j.1360-0443.2004.00540.x 14678060

[30] Yeomans K, Payne KA, Marton JP: Smoking, smoking cessation and smoking relapse patterns: A web-based survey of current and former smokers in the US. Int J Clin Pract. 2011;65. doi: 10.11 l/j.1742-1241.2011.02758.x. 10.1111/j.1742-1241.2011.02758.x 21923845

[31] Starks H, Trinidad SB (2007). Choose your method: a comparison of phenomenology, discourse analysis, and grounded theory. Qual Health Res.

[32] Kvale S (1996). InterViews: an introduction to qualitative research interviewing.

[33] Morse JM. Using Shadowed Data. Qual Health Res. 2001;11(3):291–2. 10.1177/104973201129119091. 10.1177/104973201129119091 11339074

[34] Wang IJ, Gjengedal E, Larsen T. ‘Passed and cleared’ – former tobacco smokers’ experience in quitting smoking. Glob Health Promot. 2014;21 10.1177/1757975914523480. 10.1177/1757975914523480 24603969

[35] Fowkes FJ, Stewart MC, Fowkes FG. Scottish smoke-free legislation and trends in smoking cessation. Addiction. 2008;103 10.1111/j.1360-0443.2008.02350.x. 10.1111/j.1360-0443.2008.02350.x 19032538

[36] Fong GT, Hyland A, Borland R. Reductions in tobacco smoke pollution and increases in support for smoke free public places following the implementation of comprehensive smoke-free workplace legislation in the Republic of Ireland: findings from the ITC Ireland/UK survey. Tob Control. 2006;15 10.1136/tc.2005.013649. 10.1136/tc.2005.013649 PMC2593063 16754947

[37] Wakefield M, Cameron M, Murphy M. Potential for smoke-free policies in social venues to prevent smoking uptake and reduce relapse: a qualitative study. Health Promot Pract. 2009;10 10.1177/1524839907302736. 10.1177/1524839907302736 17925593

[38] Eckerdt NS, Corradi-Webster CM. Meanings about smoking for women participant in a group for smokers. Rev Lat Am Enfermagem. 2010;18(spe):641-7. 10.1590/s0104-11692010000700022 20694436

[39] Uppal N, Shahab L, Britton J, et al. The forgotten smoker: a qualitative study of attitudes towards smoking, quitting, and tobacco control policies among continuing smokers. BMC Public Health. 2013;13 10.1186/1471-2458-13-432. 10.1186/1471-2458-13-432 PMC3651294 23641875

[40] Bommelé J, Schoenmaker TM, Kleinjan M, et al. Perceived pros and cons of smoking and quitting in hard-core smokers: a focus group study. BMC Public Health. 2014;14 10.1186/1471-2458-14-175. 10.1186/1471-2458-14-175 PMC3929905 24548463

[41] Gabble R, Babayan A, DiSante E, Schwartz R (2015). Smoking cessation interventions for youth : a review of the literature.

[42] Chan SSC, Cheung YTD, Fong DYT, Emmons K, Leung AYM, Leung DYP, Lam TH. Family-based smoking cessation intervention for smoking fathers and nonsmoking mothers with a child: a randomized controlled trials. J Pediatr. 2017;182:260-6.e4. ISSN:1097-6833. 10.1016/j.jpeds.2016.11.021 27989407

[43] Hubbard G, Gorely T, Ozakinci G, Polson R, Forbat L. A systematic review and narrative summary of family-based smoking cessation interventions to help adults quit smoking. BMC Fam Pract. 2016;17 10.1186/s12875-016-0457-4. 10.1186/s12875-016-0457-4 PMC4921023 27342987

[44] Dawson AP, Cargo M, Stewart H (2012). Aboriginal health workers experience multilevel barriers to quitting smoking: a qualitative study. Int J Equity Health.

[45] Brown J, West R. Quit success rates in England 2007-2017. Smoking in Britain. 2017;5:1-8.

[46] Ford P, Clifford A. Gussy et al: a systematic review of peer-support programs for smoking cessation in disadvantaged groups. Int J Environ Res Public Health. 2013;11 10.3390/ijerph10115507. 10.3390/ijerph10115507 PMC3863857 24169412

[47] May S, West R. Do social support interventions (“buddy systems”) aid smoking cessation? A review. Tob Control. 2000;9 10.1136/tc.9.4.415. 10.1136/tc.9.4.415 PMC1748387 11106712

[48] Caponnetto P, Keller E, Bruno CM, et al. Handling relapse in smoking cessation: strategies and recommendations. Intern Emerg Med. 2013;8 10.1007/s11739-012-0864-z. 10.1007/s11739-012-0864-z 23054409

[49] Hughes JR. Motivating and helping smokers to stop smoking. J Gen Intern Med. 2003;18 10.1111/j.1525-1497.2003.20640. 10.1111/j.1525-1497.2003.20640.x PMC1494968 14687265

[50] Abdullah AS, Ho WW. What Chinese adolescents think about quitting smoking: a qualitative study. Subst Use Misuse. 2006;41 10.1016/S0140-6736(12)60826-5. 10.1080/10826080601006433 17118813

[51] Bottorff JL, Radsma J, Kelly M, et al. Fathers' narratives of reducing and quitting smoking. Sociol Health Illn. 2009;31 10.1111/j.1467-9566.2008.01126.x. 10.1111/j.1467-9566.2008.01126.x 19055591

[52] Robson N, Bond A, Wolff K. A comparison of smoking behaviour characteristics between Caucasian smokers in the United Kingdom and Malay smokers in Malaysia. Prev Med. 2013;57 10.1016/j.ypmed.2013.04.010. 10.1016/j.ypmed.2013.04.010 23624111

[53] Oldenburg B, Glanz K, French M. The application of staging models to the understanding of health behaviour change and the promotion of health. Psychol Health. 1999;14 10.1080/08870449908407343.

[54] Bezinović P, Malatestinić Đ (2009). Perceived exposure to substance use and risk-taking behavior in early adolescence: cross-sectional study. Croat Med J.

[55] Burki SJ, Pasha AG, Pasha HA (2013). The economics of tobacco and tobacco taxation in Pakistan.

[56] Local cigarette brands being sold below legal price: The Nation. 2015. Retrieved from https://nation.com.pk/09-May-2015/local-cigarette-brands-being-sold-below-legal-price. Accessed 28 Jan 2018.

[57] Mahapatra L. The Price of Cigarettes: How Much Does a Pack Cost in Each US State? International Business Times. 2014. Retrieved from http://www.ibtimes.com/price-cigarettes-how-much-does-pack-cost-each-us-state-map-1553445. Accessed 28 Jan 2018.

[58] Tobacco Manufacturers’ association. UK cigarette price [n.d.]. Retrieved from http://globalcigarettebrands.com/uk-cigarette-prices-tobacco-manufacturers-association/. Accessed 28 Jan 2018.

