# Supplementary material for: Experiences of young smokers in quitting smoking in twin cities of Pakistan: a phenomenological study
Source: BMC Public Health. 2018 Apr 10;18:466. doi: 10.1186/s12889-018-5388-7 (PMC5891956; doi:10.1186/s12889-018-5388-7)
Supplement: Supplementary file 1 — Streubert’s procedural steps of phenomenology. Methodological steps followed during this study adapted from ‘Streubert’s procedural steps of phenomenology’. (DOCX 13 kb) [file 12889_2018_5388_MOESM1_ESM.docx]

**Table 1: Streubert’s procedural steps of phenomenology**

| **Sr. No.** | **Steps** |
| --- | --- |
| 1. | Explanation of personal description of phenomenon of interest i.e. quitting experience of smoking |
| 2. | Bracketing of presuppositions of phenomenon |
| 3. | Interviewing participants |
| 4. | **Intuiting:** Cautiously reading the interview transcripts for getting a general idea of the phenomenon experienced |
| 5. | **Phenomenological analyzing:** Reviewing transcripts to discover the real meaning (generating codes, themes/essences) |
| 6. | Understanding important relationships (between themes and essences) |
| 7. | Developing formalized descriptions of the phenomenon |
| 8. | **Literature control:** Re-evaluating the related literature |
| 9. | Distributing findings |
