# Supplementary material for: Experiences of young smokers in quitting smoking in twin cities of Pakistan: a phenomenological study
Source: BMC Public Health. 2018 Apr 10;18:466. doi: 10.1186/s12889-018-5388-7 (PMC5891956; doi:10.1186/s12889-018-5388-7)
Supplement: Supplementary file 2 — ‘Example of a part of the analysis process. An example of the analysis process: From transcript to essence. (DOCX 13 kb) [file 12889_2018_5388_MOESM2_ESM.docx]

**Table 2: Example of a part of the analysis process to formulate an essence**

| **Parts of transcript (original)** | **Parts of transcript (translated)** | **Codes** | **Themes** | **Essence** |
| --- | --- | --- | --- | --- |
| Jinho ne peena hy unho ne peena hy… jo log hyn na jo puri surrounding hy, jo puri society hy na, as society us ko bura nahi dekh rhe | Those who smoke will smoke. Actually, the people around, whole surroundings, in fact whole society does not consider smoking as something bad. | People would smoke anyway, no one in Pakistan thinks smoking is bad,  smoking is not a big deal,  Society does not consider smoking as bad, | Cigarette is acceptable in society, supportive environment in smoking. | Social conducive setup for smoking |
| Kisi bndey ko ap ne sarak pe chaltey hovey alcohol peetay hovey nahi dekha ho ga… cigarette her bnda her jaga peeta hy, kiyun key eh koi samjhta nahi hy k yeh wo cheez hy jo k alcohol ke equal ho… | You must have not seen anybody here walking on the road while drinking alcohol…but everyone smokes everywhere, this is because nobody thinks smoking is as bad as alcohol. | Smoking is not stigmatized, smoking is common, smoking is socially accepted, alcohol is stigmatized, no one gives same importance to smoking as alcohol, no one thinks smoking is bad, smoking is normal thing | Smoking is acceptable in society, smoking is not a problem, supportive environment for smoking |  |
| jb eek awaam, sb ek cheez kr rhe hy aur ap wahan pe bilkul akelay ho k bethey ve hain … to ap phr us ko usi cheez ko attain… q k jis trf muaashra jar ha hota h, bndey ka dil krta hy, me b muaashrey k sath chalun, us se alag ho k nahi dil krta… | When everybody around you is doing something and you are sitting there alone (without doing that)…then eventually you also adopt the same thing. This is because one wants to move along with the society. Nobody would like to be isolated | Smoking is common in society, smoking is something everyone is doing, smoking is normal, smoking as societal practice,  one likes to do what society do, one wants to live with trend, no one wants to be separate from society, smoking is socially acceptable | social pressure to smoke, supportive environment in smoking, Social acceptability of smoking, |  |
